# Research progress of quercetin in infection at the maternal-fetal interface

**DOI:** 10.3389/fimmu.2026.1851268

**Published:** 2026-05-28

**Authors:** Jiena Shi, Jili Zhang, Wenjie Gong, Longhui Shen

**Affiliations:** 1 Department of Pharmacy, The Affiliated Women and Children’s Hospital of Ningbo University, Ningbo, Zhejiang, China; 2 Health Science Center, Ningbo University, Ningbo, Zhejiang, China

**Keywords:** immune regulation, infection, maternal-fetal interface, pregnancy protection, quercetin, signaling pathway

## Abstract

The maternal-fetal interface serves as the core area for immune regulation between the mother and the fetus, and its stable balance is crucial for maintaining a normal pregnancy. Immune disorders at the maternal-fetal interface caused by infection are significant contributing factors to adverse pregnancy outcomes such as recurrent miscarriage, preeclampsia, and intrauterine growth restriction. Quercetin, a flavonoid compound widely present in natural plants, demonstrates significant potential for pregnancy protection in reproductive medicine. This paper systematically reviews the mechanism of action of quercetin at the maternal-fetal interface, including regulating the function of decidual immune cells, balancing the cytokine network, targeting pathogenic signaling pathways, and enhancing the barrier function of trophoblast cells and decidual stromal cells. It also integrates the existing *in vivo* and *in vitro* experimental evidence (mainly focused on lipopolysaccharide-induced infection models) and clinical research evidence to comprehensively analyze the anti-infection characteristics and application prospects of quercetin. At the same time, the safety of quercetin application during pregnancy and the optimization direction of the administration strategy were discussed. It also looks forward to future research priorities. Therefore, this article provides new ideas and theoretical support for the prevention and treatment of infection-related pathological pregnancies.

## 1 Introduction

Infection during pregnancy severely threatens the health of both the mother and fetus during the perinatal period, and acts as a high-risk pathogenic factor for multiple pregnancy-related diseases. More critically, vertical mother-to-fetus transmission of pathogenic microorganisms can induce infection in the fetus and neonate, leading to neonatal birth defects (1).

Common infection affecting pregnant women from embryo implantation to delivery and the perinatal period include reproductive tract infection such as chorioamnionitis and endometritis. The combined incidence of intrapartum chorioamnionitis is 3.9%, and that of postpartum endometritis is 1.6% (2). The common pathogens involved include group B Streptococcus, Escherichia coli, Bacteroides, Listeria monocytogenes, Ureaplasma urealyticum, cytomegalovirus, rubella virus, etc. (1–4). Currently, clinical treatments for infection at the maternal-fetal interface primarily rely on antibiotics and immunomodulators (1). The relatively safe antimicrobial agents commonly used during pregnancy include penicillins, cephalosporins, erythromycin, azithromycin, clindamycin, nitrofurantoin, fosfomycin, etc. (5, 6). Antibiotic therapy has certain limitations and drug resistance, and is ineffective against viral infections. At present, the effectiveness of antibiotic treatment for intrauterine infections is not very satisfactory. The main reason is that even if the pathogens are eliminated, the residual intrauterine inflammation can still lead to adverse pregnancy outcomes (7). The overuse of antibiotics has led to a continuous increase in the drug resistance rate (8). Some antibiotics have a certain risk of teratogenicity and need to be avoided or used with caution (6). The synergistic effect between antibiotics and immunomodulators does not always hold, and its effectiveness depends on the characteristics of the pathogens and the anti-inflammatory properties of specific antibiotics (9). Moreover, the use of immunomodulators requires caution, taking into account the safety of the fetus (7, 10). Therefore, there is an urgent need to develop safe and effective alternative strategies.

The maternal-fetal interface is a complex and dynamic microenvironment composed of decidual immune cells, trophoblasts, decidual stromal cells, and extracellular matrix. Its core function is to establish immune tolerance to fetal semi-allogeneic antigens while maintaining defense against pathogens (11). During physiologic pregnancy, this interface achieves a dynamic balance between immune tolerance and anti-infectious defense through a sophisticated immune regulatory network (12). Invasion by pathogens such as bacteria and viruses activates pattern recognition receptor-mediated inflammatory responses, leading to excessive release of pro-inflammatory cytokines, disruption of immune cell phenotypic balance, impairment of trophoblast invasion and spiral artery remodeling, thereby leading to adverse pregnancy outcomes (1, 13).

Quercetin is a naturally abundant flavonoid widely present in onions, apples, berries, and traditional kidney-toning and anti-abortion Chinese herbs such as “Cuscuta chinensis” and “Taxillus chinensis” (14). Modern pharmacological studies have confirmed that quercetin has a stable chemical structure and good biocompatibility, and its antioxidant, anti-inflammatory, and immunomodulatory activities have been verified in various disease models (15). In reproductive medicine, increasing evidence indicates that quercetin can regulate immune function at the maternal-fetal interface, reduce abortion rates, and improve pregnancy outcomes (16–18).

A comprehensive summary of the mechanism of action of quercetin in maternal-fetal interface infection and its safety during pregnancy is still lacking. This article integrates the relevant research achievements in recent years and for the first time systematically expounds the mechanism of action, research status and application prospects of quercetin in maternal-fetal interface infection, providing a reference for its clinical transformation.

## 2 Pathophysiological mechanisms of infection at the maternal-fetal interface

Trophoblast cells at the maternal-fetal interface are the principal cells constituting placental villi, and are the only embryo-derived cells that directly contact the maternal immune system. The invasion of trophoblast cells is a normal, moderate physiological process that is strictly regulated not only by the maternal body but also by the intrinsic signaling pathways of trophoblast cells. Decidual immune cells refer to cells involved in or associated with immune responses at the maternal-fetal interface, including decidual natural killer cells, macrophages, dendritic cells, T lymphocytes, myeloid-derived suppressor cells, among others. Abnormalities in the function or quantity of immune cells may trigger immune imbalance at the maternal-fetal interface, leading to insufficient trophoblast invasion, impaired decidual vascular remodeling, and reduced maternal-fetal immune tolerance, etc. During blastocyst adhesion and implantation, stromal cells in the endometrial lamina propria undergo decidualization into glycogen- and lipid-rich decidual stromal cells under the action of estrogen and progesterone. Decidual stromal cells surround the embryo and establish direct contact with it. Through secreting hormones, various cytokines and chemokines, they interact with immune cells and trophoblast cells, and play critical roles in embryonic implantation, placental development, regulation of local immune responses, and maintenance of maternal-fetal immune tolerance (11, 18, 19).

Infection at the maternal-fetal interface destroys immune homeostasis through multiple pathways and induces pathological changes in pregnancy. Pathogens can bind to pattern recognition receptors (such as Toll-like receptors, TLRs) on the surface of immune cells and trophoblasts, triggering excessive inflammatory responses. Specifically, large amounts of pro-inflammatory factors including tumor necrosis factor-α (TNF-α), interleukin-6 (IL-6), and interferon-γ (IFN-γ) are produced, which can induce trophoblast apoptosis and inhibit their invasion and migration abilities (1, 11, 13).

Infection has specific effects on the phenotype and function of immune cells at the maternal-fetal interface: decidual natural killer (dNK) cells, the most abundant immune cells at the interface, exhibit low cytotoxicity and promote trophoblast invasion under normal conditions (20), but the stimulation by pathogens can upregulate the expression of activating receptors (such as NKG2D) and enhance their cytotoxicity against trophoblasts (21); macrophages polarize toward the pro-inflammatory M1 phenotype and suppress the anti-inflammatory M2 phenotype after infection, further exacerbating the inflammatory cascade (22); meanwhile, infection disrupts the balance between T helper 1 (Th1), Th2, Th17, and regulatory T cells (Treg), shifting toward a Th1/Th17-dominant phenotype, which is unfavorable for fetal survival (23).

Trophoblasts and decidual stromal cells constitute the structural and functional barrier of the maternal-fetal interface. Infection can damage the trophoblast barrier by reducing the expression of tight junction proteins, increasing cell permeability, and inducing cell apoptosis (24). Decidual stromal cells undergo senescence or dysfunction under the stimulation by pathogens, and their ability to regulate immune cell differentiation via cytokine secretion is impaired, further aggravating immune imbalance (25). These pathological changes collectively form a microenvironment unfavorable for fetal development, ultimately leading to adverse pregnancy outcomes such as miscarriage and preeclampsia.

## 3 Mechanisms of quercetin in regulating the maternal-fetal immune microenvironment

**Figure 1:**
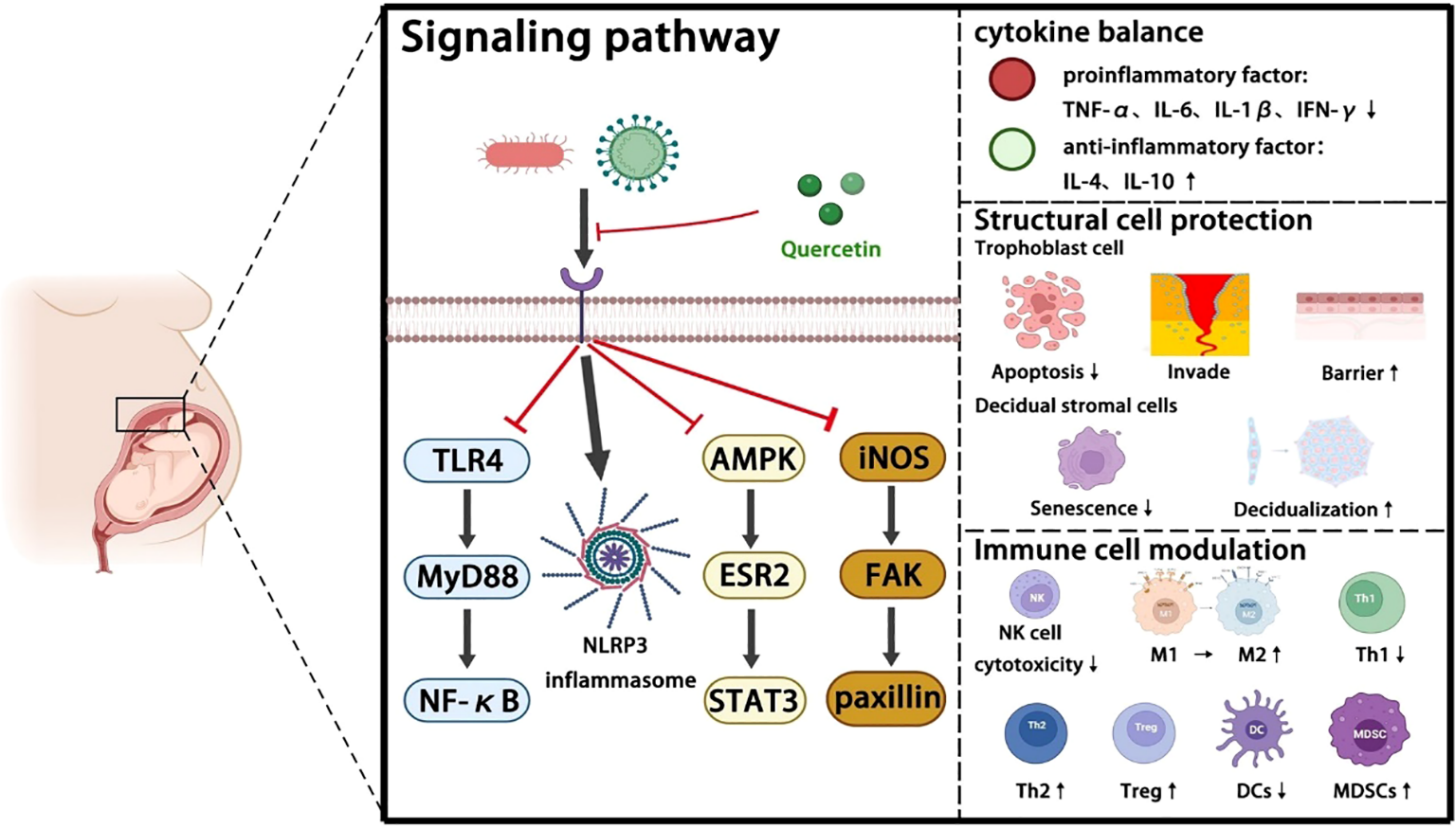
The mechanism of quercetin in regulating the maternal-fetal immune microenvironment.

Quercetin regulates the microenvironment at the maternal-fetal interface through multiple targets and pathways, including modulating the functions of decidual immune cells, balancing the cytokine network, targeting pathogenic signaling pathways, and enhancing the barrier function between trophoblast cells and decidual matrix cells. It exerts protective effects against infection-induced damage. These complex mechanisms can be summarized in the network shown in **Figure 1**.

### 3.1 Regulation of immune cell functions at the maternal-fetal interface

Quercetin plays a role in regulating the functions of immune cells at the maternal-fetal interface, including reducing the cytotoxicity of decidual natural killer cells, balancing the polarization of decidual macrophages, regulating the Th1/Th2/Treg balance of T lymphocytes, and influencing the functions of dendritic cells and myeloid-derived suppressor cells.

#### 3.1.1 Decidual natural killer cells

Functional homeostasis of dNK cells is central to the maternal-fetal immune tolerance. Indoleamine 2,3-dioxygenase (IDO) activity modulates dNK cell function (21). Increasing the expression of IDO can promote the transformation of peripheral blood NK cells into dNK cells (26). *In vitro* experiments show that quercetin significantly upregulates IDO activity in human umbilical cord mesenchymal stem cell co-culture systems, suppresses TNF-α/IFN-γ-induced inflammatory responses in peripheral blood mononuclear cells, and maintains the low cytotoxicity state of dNK cells (27). Notably, the regulation of dNK cells by quercetin is microenvironment-dependent (28).

#### 3.1.2 Decidual macrophages

The M1/M2 polarization balance of macrophages is crucial for the maternal-fetal interface homeostasis (22). Quercetin exerts multiple regulatory effects on decidual macrophages. Firstly, it inhibits macrophage activation and migration, down-regulates the expression of CD14 on macrophages in a mouse abortion model induced by lipopolysaccharide (LPS), reduces their activation and the secretion of pro-inflammatory factors, and simultaneously suppresses the production of monocyte chemoattractant protein (MCP)-1, thereby blocking the excessive recruitment of macrophages to the endometrium (29–31). Secondly, it modulates macrophage polarization: quercetin inhibits M1 polarization of human macrophages *in vitro* by regulating energy metabolism and enhances the antioxidant capacity of M2 macrophages (32). Thirdly, it prevents macrophage apoptosis: quercetin inhibits NLRP3 inflammasome activation via the TLR2/myeloid differentiation factor (MyD) 88/nuclear factor (NF)-κB pathway and reactive oxygen species/adenosine monophosphate-activated protein kinase (AMPK) pathway, thereby preventing apoptosis of human macrophages *in vitro* (33) and avoiding adverse pregnancy outcomes such as miscarriage caused by excessive inflammatory stimulation. Fourthly, it regulates macrophage number: in the LPS-induced mouse abortion model, quercetin treatment significantly reduces uterine macrophage count and lowers abortion rate (34).

#### 3.1.3 T lymphocytes

Th1/Th2/Th17/Treg balance is the core of maternal-fetal immune tolerance. Quercetin regulates T cell function through multiple pathways: in the LPS-induced mouse abortion model, it reduces the ratio of CD80+/CD86+ co-stimulatory molecules, shifting the maternal-fetal interface toward a Th2 phenotype, thus suppressing the maternal immune attack against the embryo (35); In *in vitro* human T-cell experiments, it downregulates the expression of key transcription factors in Th1 cells by inhibiting T-bet deubiquitination mediated by ubiquitin-specific protease (USP) 10, alleviating Th1-type inflammatory responses (36); *In vitro* cell experiments derived from mice and humans, it upregulates the level of myeloid-derived suppressor cells, promotes their secretion of T cell inhibitory factors, and indirectly enhances the immunosuppressive effect of Treg cells (37); It modulates T cell cytokine levels, inhibits Th1-type inflammation in the LPS-induced mouse abortion model, reduces serum TNF-α and nitric oxide levels, increases IL-10 levels, suppresses the TLR4/NF-κB signaling pathway, exerts anti-inflammatory effects, and stabilizes Th1/Th2 balance (38).

#### 3.1.4 Dendritic cells and myeloid-derived suppressor cells

Dendritic cells (DCs) mainly exist in an immature phenotype at the maternal-fetal interface and maintain immune tolerance by inducing Treg cell differentiation (39). *In vitro* cell experiments using mouse-derived samples demonstrate that quercetin reduces the secretion of pro-inflammatory factors such as IL-1β, IL-6, and IFN-γ by mature plasmacytoid DCs and myeloid DCs, preserving their immunomodulatory properties (40). Meanwhile, it downregulates intracellular iron content by regulating DC iron metabolism genes, weakening their pro-inflammatory activity of bone marrow-derived dendritic cells induced by LPS (41).

Myeloid-derived suppressor cells (MDSCs) maintain maternal-fetal interface immune tolerance by inhibiting DC and T cell proliferation and promoting Treg cell production in the LPS-induced mouse model (42). *In vitro* cell experiments derived from mice and humans, quercetin promotes granulocytic MDSC survival and enhances their immunosuppressive function via the ESR2/STAT3 signaling pathway, contributing to immune homeostasis at the maternal-fetal interface (37).

### 3.2 Balancing the cytokine network and inhibiting pathogenic signaling pathways

Infection-induced cytokine imbalance is a key step in maternal-fetal interface injury (1). Quercetin restores cytokine balance through bidirectional regulation (31, 38, 43, 44). Both the LPS-induced mouse abortion model and the LPS-induced human *in vitro* cell experiments have confirmed that quercetin can dose-dependently reduce the expression of pro-inflammatory factors such as TNF-α, IL-6, IL-1β, and IFN-γ. In terms of promoting anti-inflammatory factors, quercetin increases the secretion of IL-4 and IL-10, antagonizes pro-inflammatory factors, and facilitates immune tolerance.

Quercetin exerts anti-inflammatory effects by targeting key molecules in the following pathways (33, 37, 45). In *in vitro* human cells assay, it downregulates TLR4 and MyD88 expression in the TLR4/MyD88/NF-κB signaling pathway. In *in vitro* human cells assay, it alleviates NLRP3 inflammasome activation via the AMPK pathway. In *in vitro* cell experiments using mouse-derived and human-derived samples, it maintains maternal-fetal interface immune homeostasis through the ESR2/STAT3 pathway. In murine-derived *in vitro* cellular assay, it exerts anti-inflammatory effects by inhibiting the iNOS/FAK/paxillin pathway.

### 3.3 Enhancing the functions of trophoblasts and decidual stromal cells

Trophoblasts and decidual stromal cells form the structural barrier of the maternal-fetal interface, and their functional integrity is essential for defense against infection. Quercetin enhances their functions through multiple mechanisms. In *in vitro* human cell experiments, it protects trophoblasts cells from injury by inhibiting the activation of p38 MAPK and JNK, and reducing cell apoptosis (24). In *in vitro* human cell experiments (BeWo cells), it improves mitochondrial function and enhances the viability of trophoblast cells (46). In *in vitro* human cell experiments, it regulates the invasion of trophoblast cells by reducing the glucose uptake of HTR-8/SVneo cells, inhibiting excessive invasion of trophoblast cells, and avoiding tissue damage (47). In an experiment on pregnant mice, it also improves the function of decidual stromal cells by increasing the estradiol level in pregnant mice, activating the Wnt/β-catenin pathway, reducing the apoptosis of decidual stromal cells, and improving uterine receptivity (48). Meanwhile, in *in vitro* human cells assay, it reduces the number of senescent decidual stromal cells, increases the expression of decidualization markers (IGFBP1, prolactin), and promotes the decidualization process (49).

## 4 Antibacterial and antiviral activities of quercetin

Quercetin exhibits broad-spectrum antibacterial and antiviral activities. It has been verified that quercetin can inhibit the replication of multiple viruses, including influenza A virus, hepatitis B virus, hepatitis C virus, Ebola virus, dengue virus, herpesvirus, human immunodeficiency virus, Zika virus and SARS-CoV-2, among others (50–55). Similarly, studies have also confirmed the multi-target antibacterial efficacy of quercetin against a variety of pathogens, including Staphylococcus aureus, Pseudomonas aeruginosa, Proteus, Escherichia coli and Acinetobacter baumannii, etc. (56, 57). Recent studies have also verified its activity against multidrug-resistant bacteria. Quercetin and its metabolites can block the adhesion and invasion of methicillin-resistant Staphylococcus aureus (MRSA) to host cells and its biofilm formation, while inhibiting toxin secretion and hemolytic activity (58). In addition, quercetin may counteract multidrug-resistant Stenotrophomonas maltophilia by regulating host immune responses (59).

Although quercetin possesses broad-spectrum antibacterial and antiviral effects, existing studies on its application in maternal-fetal infection have not involved specific pathogens, and mainly focus on lipopolysaccharide-induced infection models.

## 5 *In vitro* and *in vivo* studies of quercetin in lipopolysaccharide-induced infection models

Accumulated evidence has demonstrated that lipopolysaccharide (LPS) is the predominant pathogen-derived molecule employed to establish *in vitro* and *in vivo* models of quercetin against maternal-fetal infection. As an essential component of the bacterial cell wall, LPS serves as a classic inducer of inflammation at the maternal-fetal interface and is widely used to simulate the pathological processes of infectious injury.

### 5.1 *In vitro* cellular experiments

*In vitro* cell models serve as the foundation for investigating the anti-infective effects of quercetin. As shown in **Table 1**, quercetin at varying concentrations exhibits dose-dependent regulatory effects on the expression of inflammatory factors and activation of signaling pathway proteins induced by LPS. In LPS-stimulated RAW264.7 macrophages, quercetin at 25 and 50 μmol/L significantly inhibited NO release (P < 0.05). Meanwhile, quercetin at 5, 15, and 25 μmol/L markedly reduced the mRNA expression of TNF-α, IL-6, IL-1β, iNOS, COX-2, MCP-1, TLR4, and MyD88 (P < 0.05), demonstrating a concentration-dependent manner (43). Quercetin exerts a certain effect on the M1 phenotypic polarization of LPS-induced macrophages, driving their polarization toward the anti-inflammatory M2 phenotype (32). Furthermore, quercetin inhibits pyroptosis in THP-1 macrophages by reducing NLRP3 expression and IL-1β levels in a concentration-dependent manner (33). Quercetin can reduce the secretion of inflammatory cytokines and antigen presentation by dendritic cells after LPS exposure, and downregulate genes related to iron metabolism (41). An *in vitro* study conducted by Wang et al. revealed that quercetin at 1, 5, and 10 μmol reduces the incidence of spontaneous abortion by modulating inflammatory responses and apoptosis pathways in LPS-exposed HTR-8/SVneo cells (60).

**Table 1:** Regulatory effects of quercetin on LPS-induced *in vitro* cellular experiments.

Experimental cells	LPS concentration	Quercetin concentration	Detection indicators	Results (compared with LPS model group)	References
RAW264.7 macrophages	2μg/ml	5/15/25μmol/L	mRNA levels of TNF-α、 IL-6、IL-1β、iNOS、COX-2、MCP-1、TLR4、MyD88	Significantly downregulated in a dose-dependent manner	(43)
		25/50μmol/L	NO production	Significantly downregulated in a dose-dependent manner	
THP-1 macrophages	100ng/mL	60μmol	M1, M2 metabolic components	Inhibition of M1 and enhancement of M2	(32)
THP-1 macrophages	0.1μg/ml	10/20/50μmol/L	IL-1β secretion and NLRP3 expression	Significantly downregulated in a dose-dependent manner	(33)
dendritic cell	1μg/mL	25μmol	Iron homeostasis-related genes and inflammatory factors	downregulated	(41)
HTR-8/SVneo trophoblast cells	0.2μg/ml	1/5/10μmol	IL-1β、TNF-α 、IL-6	Significantly downregulated	(60)
			Cell viability	Enhanced	

### 5.2 *In vivo* animal experiments

The mouse abortion model is a classic *in vivo* model for investigating the pregnancy-protective effects of quercetin. Quercetin can significantly improve pregnancy outcomes in model animals and downregulate the expression levels of inflammatory cytokines in uterine tissues, as shown in **Table 2**. Quercetin can prevent the migration of macrophages to the endometrium in LPS-induced mouse abortion models by inhibiting the production of MCP-1 and down-regulating the expression of CD14 (29, 31). Quercetin can regulate the local immune microenvironment of the uterus in LPS-induced abortive pregnant mice, reduce the IFN-γ/IL-4 ratio, and decrease the number of uterine macrophages, thereby playing a certain role in promoting pregnancy and protecting the fetus (34). Quercetin repairs uterine structural damage induced by LPS in the mouse abortion model, accompanied by an increase in IL-10 level and a decrease in TNF-α level (38). Quercetin exerts a tocolytic effect in the mouse abortion model, which leads to a decreased abortion rate, an increased IL-10 level, and a reduced CD80+/CD86+ ratio. All these changes are statistically significant compared with the LPS-induced abortion group (P < 0.05) (35). A study conducted by Wu et al. using an LPS-induced mouse abortion model demonstrated that intragastric administration of quercetin potentially reduces the embryo loss rate (61). In an LPS-induced mouse model of preterm birth, oral administration of quercetin reduces the incidence of preterm birth and improves neonatal survival rate (62).

**Table 2:** Regulatory effects of quercetin on LPS-induced abortion in mouse models.

Animal model	Modeling method	Quercetin administration protocol	Detection indicators	Results (compared with LPS model group)	References
Mouse abortion model	Intravenous injection of 0.5μg/ml LPS on gestational day 7	Intragastric administration of 2.5mg/ml quercetin on gestational days 2–7	Expression of MCP-1 and CD14	Significantly downregulated	(29, 31)
Mouse abortion model	Intravenous injection of 0.1μg LPS on gestational day 7	Intragastric administration of 5mg quercetin on gestational days 4–7	Abortion rate, macrophage count, IFN-γ/IL-4 ratio	decreased abortion rate, reduced macrophage count, reduced IFN-γ/IL-4 ratio	(34)
Mouse abortion model	Intraperitoneal injection of 1μg/ml LPS on gestational days 4–5	Intraperitoneal injection of 2.5mg/ml quercetin on gestational days 1–3	IL-10, TNF-α, uterine histopathology	IL-10 upregulated, TNF-α downregulated, uterine structural damage alleviated	(38)
Mouse abortion model	Intraperitoneal injection of 0.1μg LPS on gestational day 7	Intragastric administration of 0.4ml quercetin on gestational days 4–7	Abortion rate, IL-10 level, CD80^+^/CD86^+^ ratio	Abortion rate decreased, IL-10 increased, CD80^+^/CD86^+^ ratio decreased	(35)
Mouse abortion model	Intravenous injection of 50μg/ml LPS on gestational day 7	Intragastric administration of 0.25/1.25/2.5mg/ml quercetin on gestational days 3–8	Embryo loss rate	Reduced	(61)
Mouse preterm birth model	Intravenous injection of 50μg/kg LPS on gestational day 15	Intragastric administration of 30/90/150mg/kg quercetin on gestational day 15	Preterm birth incidence	Reduced	(62)

## 6 Clinical research of quercetin

To date, no clinical studies have specifically investigated quercetin for the direct treatment of human maternal-fetal infection. Furthermore, available data on the application of quercetin during human pregnancy remains extremely limited. We have summarized existing studies on the effects of quercetin on human pregnancy or human infection, as shown in **Table 3**.

**Table 3:** Clinical studies of quercetin in human pregnancy or human infection.

Subjects	Study design	Outcomes	References
Patients with recurrent spontaneous abortion	Modified Shoutai Pill (containing quercetin) combined with progesterone	The research group’s effective rate was 90.48%, higher than control’s 75.44%	(63)
Patients with recurrent spontaneous abortion	Yiqi Bushen Pill (containing quercetin) combined with progesterone	The research group’s fetal protection rate was 93.88%, higher than the control’s 79.17%	(64)
480 patients with recurrent spontaneous abortion	Combined therapy of quercetin-containing TCM compound and dydrogesterone	Reduced early abortion rate	(16)
42 COVID-19 outpatients	Quercetin administration (500mg/d in the first week, 1000mg/d in the second week)	Reduced hospitalization rate, hospital stay, requirement for non-invasive oxygen therapy and mortality	(65)
60 patients with severe COVID-19	Daily treatment with 1000mg quercetin combined with antiviral drugs for 7 days	Significant downregulation of inflammatory markers (TNF-α, IL-1β, IL-6, etc.)	(66)

Existing clinical studies on quercetin in pregnancy mainly focus on traditional Chinese medicine (TCM) compound preparations containing quercetin for patients with recurrent spontaneous abortion (RSA). These studies have verified a notable reduction in the abortion rate (9, 63, 64). Quercetin is identified as the core active ingredient of these TCM formulations; however, limitations include small sample sizes and heterogeneity in herbal compatibility among different prescriptions.

A clinical trial involving 480 RSA patients demonstrated that combined treatment with quercetin-containing TCM compounds and dydrogesterone achieved a better efficacy in reducing the early abortion rate than dydrogesterone monotherapy. The protective effect on trophoblast cells is considered a potential underlying mechanism, with quercetin serving as one of the bioactive components (16).

Clinical research on quercetin against human infection is mainly concentrated on coronavirus disease 2019 (COVID-19). In 42 outpatients with COVID-19, two-week quercetin treatment (500-1000mg daily) significantly reduced hospitalization rate (-68.2%), length of hospital stay (-76.8%), demand for non-invasive oxygen therapy (-93.3%), and mortality (65). A 7-day randomized controlled trial (RCT) enrolling 60 severe COVID-19 patients received daily combined treatment of 1000mg quercetin and antiviral drugs. The levels of inflammatory markers including TNF-α, IL-1β and IL-6 were significantly decreased in the treatment group (66).

## 7 Safety of quercetin application during pregnancy

Multiple animal studies have indicated the potential protective effects of quercetin in the maternal-fetal environment. The aforementioned LPS-induced animal experiments demonstrate that quercetin reduces the rate of embryonic loss and the incidence of preterm birth in mice, while improving neonatal survival (35, 61, 62). An early study reported that administration of quercetin (333 mg/kg) to mice did not cause any teratogenic effects on fetuses (67). Quercetin pretreatment prior to psychogenic traumatic stress alleviates anxiety-like behaviors and hematological changes in pregnant rats without altering the hematological status of neonates (68). Furthermore, maternal quercetin intake during pregnancy in mouse models attenuates fine particulate matter-induced colonic oxidative stress, inflammation, and tight junction damage in offspring (69). Maternal administration of 50 mg/kg quercetin during gestation prevents adult offspring from developing hyperglycemia, insulin resistance, obesity and hypertension induced by maternal high-fat diet (70). Another mouse study showed that maternal intake of a diet containing 0.2% quercetin during pregnancy prevents renal disease in offspring (71).

However, inconsistent results reported in some studies suggest that quercetin may also induce side effects. For instance, it has been found that quercetin at a dose of 50 μmol impairs mouse embryonic development, leading to a 20-30% reduction in the formation rate of morulae and blastocysts (72). Additionally, adult offspring of mice exposed to quercetin (302 mg/kg) during pregnancy exhibit a 40% increase in hepatic iron storage, accompanied by altered cytokine expression and hypermethylation of DNA repeats (73). Quercetin at 10 mg/kg/d affects uterine morphology without influencing proliferation; however, at a dose of 100 mg/kg/d, quercetin induces significant stromal and glandular proliferation, which may predispose the uterus to tumorigenesis (74).

Among numerous published intervention studies in non-pregnant humans, quercetin supplementation has been reported to have favorable safety profiles, and any reported adverse effects are essentially mild in nature (14, 75, 76). When used as a dietary supplement, the daily dose of quercetin can reach up to 1000 mg (76). Studies have shown that quercetin is well-tolerated in humans: no serious adverse events were reported when participants received 500 mg quercetin daily for 4–8 weeks (77, 78), 730 mg daily for 4 weeks (79), or 500 mg or 1000 mg daily for 12 weeks (80). Furthermore, all reported adverse effects are associated with a single oral dose exceeding 4g (81).

According to the U.S. Food and Drug Administration (FDA), quercetin is generally recognized as safe (GRAS) (82).

Studies on quercetin use in pregnant humans are extremely limited. The aforementioned individual clinical trials (63, 64) have shown that traditional Chinese medicine (TCM) compound preparations containing quercetin achieve favorable fetal preservation rates in patients with recurrent spontaneous abortion, with no adverse events reported. Nevertheless, the quercetin content in such TCM compound preparations has not been fully determined, and significant heterogeneity exists among different preparations.

It should be noted that quercetin inhibits major CYP450 enzymes, which can alter the pharmacokinetics of co-administered drugs, increase their bioavailability, and thus elevate the risk of adverse reactions (83). Quercetin may exert drug interactions with cyclosporine, pravastatin, fexofenadine, diltiazem, antihypertensive drugs and other agents (76, 84), thus caution is required when co-administering medications during pregnancy.

In addition, the toxic mechanism of quercetin is commonly referred to as the “antioxidant paradox”: at high concentrations or in the presence of transition metals, quercetin switches its activity from antioxidant to pro-oxidant (85). Quercetin impairs mitochondrial integrity by inhibiting biosynthesis, disrupting the coordination between replication and transcription, and accumulating in mitochondria (86). Quercetin has potential nephrotoxic effects, and patients with renal dysfunction may be a population at potential risk for long-term high-dose quercetin supplementation (76). Renal-specific damage was observed in long-term exposure experiments in F344/N rats (85). An acute exposure study in BALB/c mice demonstrated dose-dependent hepatorenal toxicity, and the LD50 of quercetin-rich extract was 3807 mg/kg (87).

## 8 Limitations and application prospects of quercetin in maternal-fetal infection

Currently, the application of quercetin in maternal-fetal infection still faces multiple challenges.

Quercetin exhibits low bioavailability. After oral administration, its poor water solubility and extensive metabolism in the intestine and liver lead to insufficient bioavailability. Recent progress has primarily focused on addressing these challenges through the development of novel chemical derivatives and advanced drug delivery systems, including improvement achieved via structural modifications (e.g., glycoside-sulfate conjugates) or new formulations (e.g., nanoparticles, hydrogels, liposomes) (85, 88, 89). Although preclinical research results are promising, large-scale randomized controlled trials are still required. Future research should incorporate pharmacokinetic and pharmacodynamic analyses, as well as nanotechnology-based drug delivery systems or combination drug therapy strategies.

The dose optimization of quercetin has not yet been clarified, and the conventional clinical dose is 500–1000 mg/day (77, 79, 80). Further research is needed to determine the safe and effective dose range during pregnancy.

There are significant individual differences, and genetic polymorphisms may affect the efficacy and safety of quercetin. Pharmacogenetic variations in quercetin-metabolizing enzymes (e.g., UGTs, SULTs) and transporters (e.g., OATPs, P-glycoprotein) significantly affect the systemic exposure of quercetin and its reactive intermediates *in vivo* (90–94). In the future, individualized medication strategies may be needed.

There is a lack of large-scale clinical research on quercetin targeting infections at the maternal-fetal interface during human pregnancy, and its clinical efficacy and safety require further validation.

Current research is mostly focused on LPS-induced infection models, while the mechanism of action of quercetin against infections caused by other pathogens such as viruses and protozoa remains unclear, necessitating expanded research in this area.

Quercetin interacts with a variety of drugs and has potential nephrotoxicity, therefore close medication monitoring during pregnancy is required. Existing research on the impact of quercetin on the long-term health of offspring is insufficient, and targeted studies are needed to address the aforementioned issues in future research.

## 9 Conclusion

The summarized data on quercetin indicate that it might have therapeutic potential in the management of maternal-fetal interface infection through multi-target immunomodulation, anti-inflammatory effects, and protection of cellular barrier function, although direct evidence is still lacking. Current preclinical studies have laid a solid theoretical foundation for its future clinical translation. In-depth exploration of its mechanism of action and the implementation of standardized clinical research will constitute a critical step in promoting the application of quercetin in the prevention and treatment of infection-related pregnancy complications.
